# Advances in Bio-Based Polymers for Colorectal Cancer Treatment: Hydrogels and Nanoplatforms

**DOI:** 10.3390/gels7010006

**Published:** 2021-01-11

**Authors:** Anna Maspes, Fabio Pizzetti, Arianna Rossetti, Pooyan Makvandi, Giovanni Sitia, Filippo Rossi

**Affiliations:** 1 Dipartimento di Chimica, Materiali e Ingegneria Chimica “Giulio Natta”, Politecnico di Milano, 20131 Milan, Italy; anna.maspes@mail.polimi.it (A.M.); fabio.pizzetti@polimi.it (F.P.); arianna.rossetti@polimi.it (A.R.); 2 Istituto Italiano di Tecnologia, Centre for Micro-BioRobotics, 56025 Pisa, Italy; pooyan.makvandi@iit.it; 3 Division of Immunology, Transplantation and Infectious Diseases, Experimental Hepatology Unit, IRCCS San Raffaele Scientific Institute, 20132 Milan, Italy; sitia.giovanni@hsr.it

**Keywords:** cancer, chemotherapy, colorectal, hydrogels, nanoparticles

## Abstract

Adenocarcinoma of the colon is the most common malignant neoplasia of the gastrointestinal tract and is a major contributor to mortality worldwide. Invasiveness and metastatic behavior are typical of malignant tumors and, because of its portal drainage, the liver is the closest capillary bed available in this case, hence the common site of metastatic dissemination. Current therapies forecast total resection of primary tumor when possible and partial liver resection at advanced stages, along with systemic intravenous therapies consisting of chemotherapeutic agents such as 5-fluorouracil. These cures are definitely not exempt from drawbacks and heavy side effects. Biocompatible polymeric networks, both in colloids and bulk forms, able to absorb large quantities of water and load a variety of molecules-belong to the class of innovative drug delivery systems, thus suitable for the purpose and tunable on each patient can represent a promising alternative. Indeed, the implantation of polymeric scaffolds easy to synthesize can substitute chemotherapy and combination therapies scheduling, shortening side effects. Moreover, they do not require a surgical removal thanks to spontaneous degradation and guarantees an extended and regional cargo release, maintaining high drug concentrations. In this review, we focus our attention on the key role of polymeric networks as drug delivery systems potentially able to counteract this dramatic disease.

## 1. Introduction

Adenocarcinoma of the colon is the most common malignancy of the gastrointestinal tract (GIT) and is a major contributor to mortality worldwide, while the small intestine is an uncommon site for malignant tumors even though it accounts for 75% of the overall GIT length. With 50,000 deaths/y in the US [1] and 20,000 deaths/y in Italy, colorectal adenocarcinoma is second for cancer death. Its incidence peaks at 60–70 years of age, affects males slightly more than females and is geographically dependent on dietary factors.

Its pathogenesis includes genetic and epigenetic abnormalities; indeed, colorectal cancer (CRC) often grows as polypoid masses (in the cecum and ascending colon) or as annular lesions that cause obstruction (in distal colon). The general microscopic characteristics of right- and left-sided colonic adenocarcinomas are similar, and the tumor is usually equally distributed over the entire length of the colon [2]. CRC is detectable by endoscopic screening, preceded by iron-deficiency anemia in older males and postmenopausal females detected by CBC (complete blood count). The main symptoms are occult bleeding, changes in bowel habits and cramping discomforts [1].

Total resection of the tumor is the optimal treatment when a malignant lesion is detected in the large bowel. Before and after surgery, a colonoscopy of the entire large bowel should be performed together with a CT scan and evaluation of the presence of metastatic disease through biochemical assessment of liver functions. Following recovery from a complete resection, patients should be observed carefully for five years by physical examinations and blood tests. Indeed, patients cured of CRC have a 3–5% probability of developing additional bowel cancer during their lifetime and a >15% risk of the development of further polyps.

Radiation therapy, performed either pre- or postoperatively, reduces pelvic recurrence but does not appear to prolong survival. Therefore, it is not effective as a primary treatment of colon cancer. Systemic therapy becomes more effective: 5-fluorouracil (5-FU or 5-fluoro-2,4-pyrimidinedione) administration remains the backbone treatment for this disease. The efficacy is enhanced for patients with liver metastases when chemotherapy is infused directly into the hepatic artery or portal vein, but this treatment results in toxic, costly and does not lead to appreciably survival prolongation. 5-FU administration with some adjuvants (folinic acid, irinotecan, leucovorin, oxaliplatin, paclitaxel, piroxicam) improves response rates and survival of patients with metastatic diseases; it can be administered intravenously or orally with similar efficacy (administration from the rectum is not effective in distributing the precise drug dose to the entire area of the colon [3]), producing average survival of two years. Monoclonal antibody treatments are also effective in patients with advanced CRC: avastin (cetuximab, bevacizumab or panitumumab [4]) can act as an anti-angiogenesis agent with hypertension, proteinuria and thromboembolic events as possible side effects [1].

Patients with solitary hepatic metastases without clinical or radiographic evidence of additional tumor involvement should be considered for partial liver resection; this procedure is associated with 5-year survival rates of 25–30% of patients [1].

The most suggested therapy is a combination of systemic intravenous administration of chemotherapeutic agents, the correct choice for a patient is made by biomarker analysis, but it is further complicated by the fact that current treatment guidelines for metastatic CRC differ around the world [4]. Over the past decades, advances in surgical management and identification of novel therapeutic targets have led to significantly improved survival of patients with colorectal liver and lung metastases [5]. CRC metastasis dissemination to the liver is one of the most life-threatening malignancies in humans and represents the leading cause of CRC-related mortality. In fact, only 15% or fewer of patients with colorectal adenocarcinoma at the stage of distant metastases to the liver are alive after five years from diagnosis. Due to the portal drainage, the liver is the most common site of metastatic lesions.

## 2. New Treatments for Liver Colorectal Cancer Metastases

More therapeutic options are now available than ever before for patients with metastatic colorectal cancer: the clinical decision has become even more complex since it depends on the patient’s history, cancer stage and presence of metastases [6]. If new chemotherapy strategies suggest combining biological agents to target different growth factor receptors, and despite the excess of information available, the optimal treatment strategy for patients with metastatic CRC remains unclear [4].

The main issue lies in determining the nature of the so-called tumor microenvironment (TME) for analyzing the changes that indicate spreading. The role of TME in cancer progression is increasingly recognized, and it is considered the description of the behavior of tumor stroma in terms of biological content of immune cells, lipids, proteins, nucleotides, metabolites, growth factors, cytokines and chemokines for signaling. Many of TME components are characteristic of the extracellular matrix (ECM) structure, whose analysis can assess the differences between normal colon, primary CRC and CRC with liver metastasis, as well as its changes in time for developing the target treatments. ECM is an essential and dynamic component of all tissues and directly affects cellular behavior by providing mechanical stability and biochemical signaling cues. Its changing can alter many functions and promote tumor generation: ECM from normal human colon and CRC metastasized to liver present differences in protein composition and stiffness, with significant modifications in the vascular network formation. Hence, modeling of ECM and TME can help to find the correct treatment [7]. Side effects, such as leucopenia and diarrhea, are commonly seen during combination treatments, irrespective of the biologic agent or chemotherapy regimen used; surgery also is not exempt from some drawbacks regarding the immune system. While studying TME, alternative techniques are trying to be developed solve the immune downregulation (leucopenia) and the other side effects due to chemotherapy and resection. Different scientists have tried to study and reproduce three-dimensional cell cultures able to reproduce the TME through the usage of biomaterials. In these cases, the conditions obtained were similar to the in vivo ones, thus facilitating research on cancer drug discovery [8,9].

### 2.1. Delivery Technologies for Combination Therapies

Immunotherapy, as already mentioned, is one of the newest treatments for cancer and metastases, but it still presents serious adverse effects, including autoimmunity and nonspecific inflammation, even though the off-target drawbacks are fewer than those shown with chemotherapy. This technique uses agents to activate or boost the activation of the immune system to attack cancer cells through natural mechanisms, many of which are evaded during disease progression, as previously seen [10]. The first marketed techniques are the administration of recombinant versions of the cytokine IFNα and interleukin-2 (IL-2). The latter showed a durable complete response in some patients, but the high doses requested led to many adverse effects [11,12]. The challenges to face are efficacy and safety since the responses are different from patient-to-patient, and the autoimmune deregulation can sometimes lead to attack healthy tissue [13]. Nowadays, immunotherapy is approved for hematological cancers, while it is less effective for solid tumor therapy. Therefore, there is a need to improve the delivery technologies for increasing the accumulation of the agent within the targeted tissue aiming to reduce side effects. Novel delivery platforms under current study—hereby showed—are [3,13]:

- Hydrogels [14]: implants, scaffolds, micelles, hydrogel beads or microspheres [12];
- Nanoparticles (NPs): microparticles, liposomes, solid lipid NPs [15], polymeric NPs [16], niosomes, polymersomes;
- Other biomaterials and cell-based platforms.

The approach used depends on the chemotherapeutics or immunotherapeutic agent chosen (both of them are used in combination therapies) and whether the delivery is local or targeted. The benefit achieved with these techniques with respect to the agent alone is evident: they protect the therapeutic cargo until it is delivered to the targeted cells, they show spatiotemporal control over the delivery, they inactivate the payload until needed and they localize and control delivery for drugs. Optimal results, long-lasting and non-toxic therapies were also reached for colorectal cancer specifically, as reported for instance by A. I. Matos et al. [17] and by S. Rahimian et al. [18] and with the other materials hereon described.

### 2.2. Hydrogels

Hydrogels—discovered in 1968—are a class of crosslinked polymers that, due to their hydrophilic nature, can absorb large quantities of water. These materials uniquely offer moderate-to-high physical, chemical and mechanical stability in their swollen state, depending on their specific application.

Polymers are chosen for their regulable molecular weight, tuning properties and response to stimuli. Biocompatible hydrogels can be prepared using a variety of polymeric materials, broadly divided into two categories according to their origin: natural or synthetic [19]. Natural polymers are mainly polysaccharides, perfect for hydrogels that can mimic aspects of the structural and biological properties of the cellular microenvironment: mechanical properties, water content, flexibility and their dynamic nature help resembling the natural ECM. Unfortunately, despite their optimal biocompatibility, natural polymers have limited tunability and degradation kinetics. On the other hand, synthetic polymers can include the introduction of degradable or biochemical moieties, making them excellent candidates for hydrogels in spite of their limited biocompatibility. Often blends of both are used to match the specifications [20].

Hydrogels are usually defined by their degree of swelling, determined by the amount of water uptake inside their network and the nature of polymer–water interactions. Hydrogels with hydrophilic functional groups swell in water exclusively as a result of polymer–water interaction forces, whereas ionic hydrogels submit significant expansion in space for water absorption since charges repel one another. The balance between elastic forces and the strength of network crosslinking defines the equilibrium hydrogels swelling. The ability of a hydrogel to swell is defined by its synthesis parameters, which determine porosity and crosslinking density (distance between two crosslinks on the same polymer chain). The swelling process can be seen as a diffusion process followed by a relaxation process: the former is governed by the diffusivity rate, the latter by the relaxing rate, which is nothing but a slower absorption process. Weight, volume, turbidity and dimension values change during the swelling process: any of these factors may be used to characterize the hydrogel swelling behavior. The hydrogels’ possible payloads and consequent final shape—scaffold or beads—are many and depend on the application. Typical acellular cargos are drugs such as chemotherapeutics, proteins (interferons or other cytokines) and growth factors for enhancing regeneration, achieve a sustained release or help to signal, but also nanocarriers loaded in turn with drugs for assessing active targeting. Instead, for tissue engineering, the principal load is constituted by living cells: the hydrogel must provide the natural cell microenvironment in order to assess appreciable cell viability, namely the correct mechanical strength which resembles the ECM and cell-ECM interactions, it must allow signaling—essential for a cell to live and communicate with other cells and structures as well as to differentiate—and nutrients supplying with the possibility to get rid of wastes. In the case of cellular strategy, incorporation of biochemical cues is fundamental too for modulating cell adhesion, migration, differentiation and proliferation. The aim is to recall other cells for helping tissue healing, providing the correct environment. It is important that the cargo is well-bounded inside the hydrogel for avoiding immune system cell entrance and protecting from recognition as foreign material. The encapsulation of biochemical materials can be achieved through chemical immobilization or physical encapsulation. The former is used mainly for cell-binding, paying attention to side reactions in order to avoid cytotoxicity [21], while the latter is primarily implied in growth factors, proteins and nanoparticle-loading. Degradation, together with swelling and diffusion, is responsible for payload release. Controlled degradation of hydrogels is highly desirable for biomedical applications and depends on the final specific one [22]. It can be achieved by forming hydrogels with degradable polymer backbones, degradable crosslinks, degradable pendant groups or reversible non-covalent interactions. Too rapid degradation can lead to an initial sudden release of cargo, generating large bioactive molecule concentration which may be undesirable or even toxic. Thus, degradation must be tuned with the crosslinking density, the location of degradable moieties and the mesh size, which can limit accessibility owing to a hindered diffusion rate [20]. A controlled degradation process is essential to obtain the correct therapeutic window with a sustained release: breakage of polymer and crosslinks frees the payload; the slower the rupture, the most gradual the release.

Hydrogels can degrade through surface erosion, bulk degradation or a combination of the two. The former is favored by restricted diffusion due to high crosslinking density; the latter is due to high water content. Chemically crosslinked hydrogels can be degraded in smaller segments through several mechanisms: cleavage of the backbone chain, crosslinker or pendant groups.

The chemical reactions responsible for the process are often hydrolysis, enzymatic cleavage, reversible click reactions or photoinitiated degradation.

### 2.3. Nanoparticles

**Figure 1:**
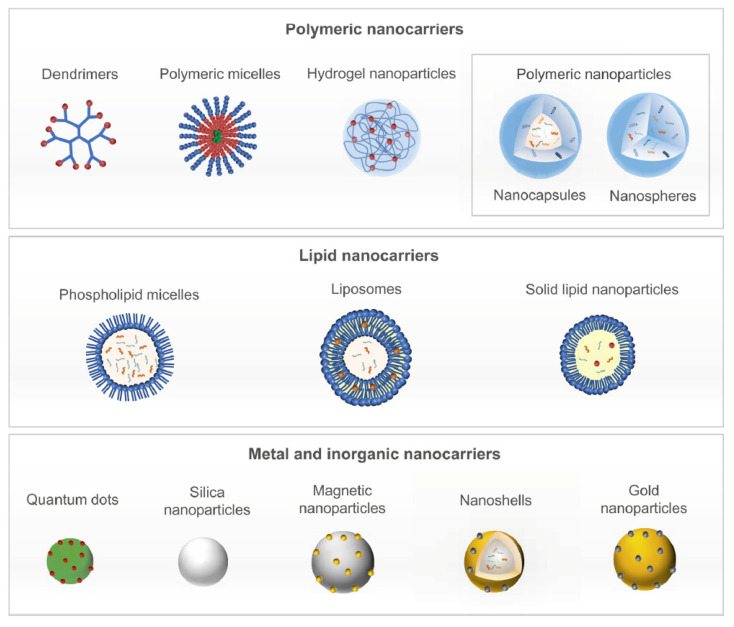
Major classes of nanoparticles (NPs) under clinical trial for cancer therapy. Reprinted with permission from Elsevier [17].

Nanomedicine is the design and development of therapeutics and diagnostic tools distinguished by the nanometric scale of its delivery vehicles and diagnostic agents with unprecedented safety and efficiency [23]. Nanotechnology deals with the manipulation of matter on the molecular scale, generally less than 1 μm. Nanoparticles (NPs) are thus minute particles, typically less than 200–100 nm in diameter, able to facilitate intracellular uptake and encapsulate therapeutic agents, releasing them in a controlled manner to specific diseased cells through passive or active targeting. The main properties of NPs are the large volume-to-surface area ratio, the size, modifiable external shell and surface properties, biodegradability, low cytotoxicity and optimization of pharmacokinetics reducing the dose [24]. The major classes of nanoparticles under study for cancer treatment are amphiphilic block copolymers that can self-assemble into micelles, polymers with pendant groups, lipid NPs that can be either solid with one layer or liposomes made of more than one wall (Figure 1) [25].

All NPs contain at least two fundamental spatial components: the core and the corona that interact with the environment or solvent (also multilayer, more complex systems exist). The possible cargos comprehend nucleic acids for gene therapy, peptides, proteins, chemotherapeutics and other drugs, but unlike hydrogels, NPs cannot carry living cells. These cargos can be shipped either in the core or on the shell, depending on their affinity with one component or the other. The nanoscale requires very specialized formulation methods. The most common syntheses employ self-assemble processes to amphiphilic lipid, polymer or polymer–drug conjugates, including nanoprecipitation, oil-in-water single emulsion and water-in-oil-in-water double emulsification [26].

**Figure 2:**
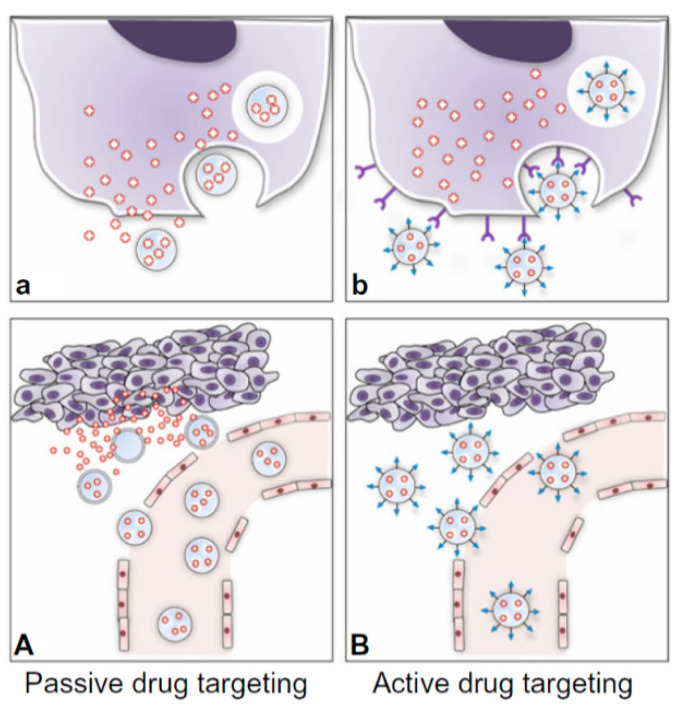
Drug targeting and localization of nanosystems. (**A**) Passive targeting through enhanced permeability and retention (EPR) effect and (**a**) drug releasing into the extracellular matrix (ECM), diffusing through the cell wall. (**B**) Active targeting with grafted ligands able to (**b**) target receptors of tumor cells enhancing uptake and internalization via receptor-mediated endocytosis. Reprinted with permission from Dove Medical Press [23].

Recent developments include the discipline of microfluidics: NPs self-assemble through a diffusive mass transfer at the interface of miscible liquids, provoking high costs of large-scale production [27,28]. Surface modification with PEG protects NPs from clearance from the blood by the mononuclear phagocytic system (MPS), markedly increasing both circulation time and drug uptake by target cells [29]. Indeed, PEG is currently the gold standard for NPs coating because it dramatically reduces protein adsorption through hydrophilicity and steric repulsion effects: this allows “stealth” NP carriers to persist in the bloodstream long enough to reach or recognize their therapeutic site of action, reducing toxicity and allowing image capture. More recent techniques involve zwitterionic polymer-based NPs, able to avoid nonspecific protein adsorption thanks to electrostatically induced hydration, with good affinity, stability and bioactivity. Zwitterionic polymers—such as poly(carboxybetaine)—have a pH-switchable moiety that alters the surface charge and allows recognition by tumor cells due to pH differences between the normal tissue and the tumor microenvironment [30,31]. The biodistribution of first-generation NPs follows the passive targeting: enhanced permeability and retention (EPR) effect refers to the fact that tumors retain more polymeric NPs, proteins, liposomes and micelles than other tissues. Tight junctions in normal vasculature prevent particles larger than 2 nm from crossing between endothelial cells, while junctions and basement membrane of tumor vasculature are disordered, allowing entities of 10–500 nm in size to extravasate and accumulate within the tumor interstitium. Moreover, the lymphatic drainage system is also impaired in cancers, further entrapping macromolecular particles and delaying their clearance. Passive targeting is based on both the minute size of drug carriers and the leaky neovasculature of the tumor [24,32]. Passive targeting has, unfortunately, several drawbacks, like not efficient delivery since particles are trapped mainly in the liver and spleen due to their reticuloendothelial function. In addition, EPR is a very heterogeneous phenomenon: its extent varies between cancers, even intratumorally, and the higher interstitial pressure within the tumor core causes the NPs to flow from the inner regions to the outer ones. Moreover, PEGylation for “stealth” purposes increases hydrophilicity on the NP surface, and this can avoid its uptake by cancer cells, thereby hindering efficient drug delivery to tumors by passive targeting [23]. Efforts are thus needed to synergize passive targeting with a more dynamic method capable of further improving the accumulation of NPs at disease sites: up to now, 90% or more of the therapeutic agent do not reach the site of action. Active targeting can be a method to achieve special localization by intentionally homing NPs to active diseased sites while eliminating off-target adverse effects in normal tissue. Polyvalent decoration of an NP’s surface with a ligand can facilitate binding to a biomarker that is specifically overrepresented in targeted cells and trigger receptor-mediated endocytosis [33]. The ligand used include antibodies, engineered antibody fragments, proteins, peptides and small molecules [23]. Active targeting is promising, but in few studies reaches the clinical trial because of the many issues faced—like different behavior in humans or protein interactions in the body which interrupt the orientation and proper display of the targeting ligand. An active targeting approach can be used for controlled drug release applications, where the drug is released into either the extracellular or intracellular compartment. In the latter process, the internalization of NPs by receptor-mediated endocytosis can occur through several pathways that lead to endosome formation and ultimately allow for the generation of lysosomes [34]. Cell internalization by active targeting NPs is postulated to improve the therapeutic efficacy compared with non-targeted NPs: passive targeting allows cargo releasing mainly in the ECM, with poor NP internalization (Figure 2).

NPs uses depend on the payload and functionalization, but here the attention has been focused on their implication in CRC diagnosis and treatment. Indeed, engineered NPs may be an optimal tool for spatiotemporal controlled delivery to overcome barriers of combination therapies [35].

Historically, Doxil^®^ was the first FDA-approved nanodrug in 1995 for general cancer therapy. It consists of a liposome with a lipid bilayer in a “liquid-ordered” phase composed mainly of cholesterol and loaded with doxorubicin; moreover, it avoids MPS recognition thanks to PEGylation. Due to the EPR effect, Doxil is passively targeted to tumors, and its chemotherapeutic cargo is released and becomes available to cancer cells only. Despite the large reward and time since the discovery, there is still no FDA-approved generic “doxil” available at a lower price [36]. Nanotechnology represents the “new era” of cancer detection and treatments: versatile payloads with favorable PK and cellular targeting for enhanced specificity, efficacy and safety [37]. Nanoparticles—like hydrogels—can also be implied for a better imaging-based diagnosis thanks to magnetic or fluorescent ligand attached, used as contrast agents [38]. For CRC specifically, it is possible to detect the stage and the presence of insurgent micrometastases with NPs ligands (heat-stable peptides) that bind a unique surface-bound receptor (guanylyl cyclase C–GCC) expressed on primary colorectal carcinomas and its metastases. Images can be taken through MRI conjugating iron oxide molecules to the structure [39]. After detection, targeted NPs could take advantage of differentially expressed molecules on the surface of CRC and liver metastases cells, providing effective release of cytotoxic drugs [40]. Another worth mentioning approach is gene therapy applied to CRC metastases: the particular genetic cargo has a therapeutic efficacy without the need for a toxic drug, even though an effective and safe system capable of exclusively targeting metastatic cancers that have spread to distant organs or lymph nodes does not exist yet. DNA or RNA (mainly) can be encapsulated as cargos or constitute a multi-arm nanoparticle themselves; ligands for active targeting, fluorophores for imaging and other drugs can be attached also in this case. RNA NPs demonstrated simultaneously receptor-targeting of CRC cancer cells and liver/lung metastases in plenty of studies [41]: the NPs are internalized causing the transcription of engineered toxic proteins that kill neoplastic cells from the inside. As with all NPs therapies, this treatment requires systemic administrations. In combination therapies, the synergistic effect of two (or more) agents—immune and chemotherapeutics—targeting different disease pathways, genes or cell-cycle checkpoints in the cancer process is increasingly used to raise the chances of eliminating cancer; dissimilar pharmacokinetics and biodistribution of agents complicate this task.

Nanoparticles provide opportunities for designing and tuning properties that are not possible with other types of therapeutics, making them a proven class of treatments for cancers. However, they are not exempt from issues of concern: how nanoscale behaves in humans and how NPs move through tumor tissue once they have localized into the neoplastic area are still opened questions. The ideal nanocarrier for cancer therapy should be “personalized” and should fit the following profile [34]:

- Biodegradable and biocompatible;
- Capable of effective homing with most of the therapeutic agent localized within the target site;
- Optimal properties design for superior drug-loading, circulation, half-life and sustained drug release across infrequent administration times;
- Affordable, cost-effective scale-up for commercialization.

### 2.4. Biomaterials for the Treatment of CRC and Liver Metastases

After this brief overview of hydrogels and nanoparticles’ main properties and applications, different tested materials with their payloads and therapeutic results are introduced for the specific treatment of liver metastasis, a major problem of CRC.

#### 2.4.1. PEG/PLA

Block copolymers consisted of poly(ethylene glycol) (PEG) and biodegradable polyesters, such as poly(lactic acid) (PLA), have been developed to obtain biodegradable and biocompatible thermogelling hydrogel for drug delivery [42]. The most used composition is PLA–PEG–PLA triblock copolymer, but lactide has three stereoisomeric forms, D-lactide (PDLA), L-lactide (PLLA) and meso or D,L-lactide, depending on the orientation of methyl groups; the principal shapes used are PDLLA–PEG–PDLLA (PLEL) or PEG–PDLLA, obtained with a stereocomplexation of the two enantiomers. The A–B–A copolymer consists of hydrophilic PEG and hydrophobic polyester blocks, hence showing amphiphilicity in aqueous solution with the length of the blocks that vary the affinity: polyester-polyether block copolymers could self-assemble into core-shell-like micelles in water. The A-B copolymer forms similar nanovesicles with hydrophilic surfaces and hydrophobic inner structure. The basic shape used is that of injectable thermosensitive hydrogel: the aqueous PDLLA–PEG–PDLLA solutions above critical gel concentration could reversibly transform into non-flowing non-crystalline hydrogel spontaneously within 2 min around the body temperature either in vitro or in vivo, following the micellar mechanism mentioned above. Sol–gel transition behavior, amphiphilicity and the mechanical properties of this material can be adjusted, modulating the molecular weight, block length and polymer concentration. Gelling, together with the one-step facile and safe synthesis (ring-opening polymerization of D,L-lactide with PEG as initiator and yield of 90%), enables to incorporate of pharmaceutical agents simply by mixing in the sol state followed by the injection in the targeted tissue [43]. Nanovesicles of PEG–PDLLA are instead injected directly in the blood circulation and are able to maintain their shape for all the journey, exploiting the EPR effect of cancers for achieving passive targeting and selecting mainly liver metastases. The core can act as a store for the controlled release of hydrophobic drugs, such as light-sensible hematoporphyrin monomethyl ether (HMME), together with the shell, which can encapsulate hydrophilic chemotherapeutics and bioactive agents, like doxorubicin (DOX) [44]. The process of synthesis involves a double emulsion method for englobing both compounds. In addition to DOX, an alternative therapeutic cargo is a curcumin (Cur) that effectively inhibits the generation and metastases of colon carcinoma, while active targeting can be achieved by conjugating the ligand folate (Fol) to the free moiety of PEG [45,46]. Folate (or folic acid) is a small molecule classified as the ligand required for essential cell function and which has the ability to link closely with highly expression folate receptors on the surface of many cancer cells.

**Figure 3:**
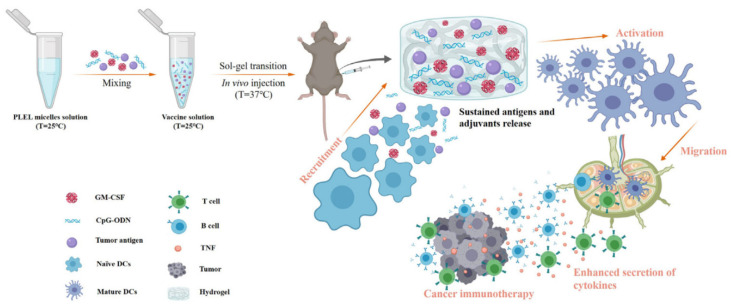
In vivo immune regulation mediated by the hydrogel vaccine. Reprinted with permission from Elsevier [47].

The newest use of this material—still currently under testing and improvement—involves immunotherapy: a PLEL reversible thermosensitive hydrogel for *vaccine* encapsulation. In this application, the cargo is composed of cytokines—involved in immune regulation, leukocyte proliferation and DCs activation—together with Toll-like receptor agonists aimed to activate antigen-presenting dendritic cells. The solution proposed, as soon as injected, forms a hydrogel scaffold thanks to micelles packing, releasing slowly and continuously antigens and immunomodulators able to locally recruit, activate and maturate DCs (Figure 3). The potential immunogenic environment created is demonstrated to eradicate colorectal cancer, emptying its cargo in seven days through a swelling diffusion process, which delays hydrogel degradation [47].

The hydrogel variant with PLLA only is not suitable for encapsulation of proteins and some drugs due to the higher temperature to ensure stability. Hydrogel injection at elevated temperatures is uncomfortable for patients; PDLLA_1500_–PEG_1500_–PDLLA_1500_ instead was chosen as the best option. Degradation experiments illustrated that the physical hydrogel could retain its integrity for several weeks and eventually be degraded by hydrolysis and enzyme-assisted methods. Little cytotoxicity and hemolysis (below 5%) of this polymer were found, but the inflammatory response in vivo after the hydrogel injection was acceptable, drawing it as a safe material. Cytotoxicity is exhibited first, like acute inflammation, which becomes mild chronic and lasted for 6 weeks; 10 weeks later, the tissue was analyzed as normal, and the hydrogel is completely disappeared [43]. In vivo tests showed that the empty micelle is suitable to counteract postoperative peritoneal adhesions and bowel surgery abrasions, but the difficulty to reach in situ injection for CRC and its liver metastases are worth mentioning. Nanovesicles instead avoid drug resistance and multiple side effects of chemotherapeutic drugs—as cardiac toxicity, myelosuppression and damages to GIT mucosal cells—and the synergistic effect of the double load can produce reactive oxygen species able to kill specifically liver tumor cells, with low toxicity to normal tissues. This method can help to solve the problem of unsatisfactory targeting of chemotherapeutics—stability of Cur, for instance—and poor water solubility of photosensitizers, which otherwise would undergo the natural opsonization process before targeting the CRC or its metastases. In particular, the Fol ligand conjugation improves the targeting ability of the system and the retention time in the body, enhancing specificity and efficacy of the treatment. PDLLA–PEG nanovesicles containing DOX are known on the market as Genexol-PM^®^ since 2007. The alternative application of PLEL hydrogels for immunotherapy allows overcoming the in situ injection problem. The gel has the only task to deliver cytokines to trigger dendritic cell activation, which will then migrate to lymph nodes and boost T cells differentiation, no need to reach the tumor site with a syringe needle. Results state that the survival of CRC patients is increased by 20% after 30 days of therapy due to the production of TNF cytokine secreted by activated immune cells that can directly kill cancers.

Prolonging the residence time of antigens and adjuvants through a scaffold stimulates the production of a stronger and more persistent immune response, but the mechanism of tumor elimination is not well understood yet. Current studies are also assessing the efficacy of hydrogel combination therapies on primary and CRC-derived hepatocellular carcinoma.

#### 2.4.2. PEG/PCL

Another biodegradable and biocompatible block copolymer made of PEG and polyesters, poly(ε-caprolactone) (PCL), is presented here. Often this confirmation is seen as a better variation of Pluronic^©^: using PCL instead of polypropylene glycol (PPG) can solve the weak hydrophobicity of the latter, resulting in a distinct decrease in molecular weight after degradation and easier elimination from the body, owing to an evident decrease in CMC [48]. PCL is a biodegradable, non-toxic and FDA-approved polymer with great permeability. Its monomer is ε-caprolactone, a cyclic ester possessing a seven-membered ring. The copolymers PEG/PCL-based implied in drug delivery technologies have different blocks’ organization: from the basic PEG–PCL diblock copolymer (PECL) [49] to the variants of the triblock copolymer, that is PEG–PCL–PEG (PECE) [50] or PCL–PEG–PCL (PCEC). Compared with PECE copolymers, PCEC has many advantages, namely the one-step synthesis without needing toxic coupling agents, wider gel window and longer persistence in vivo (around two weeks) [51]. These copolymers are synthesized by ring-opening polymerization of the lactone in the presence of PEG when the injectable hydrogel form is needed or through emulsion solvent evaporation method when nanoparticles shape is preferred. The resulting product had been tested as non-toxic, and with no histopathological change occurrence, thus it could be indicated as a safe candidate for application in biomedical fields. Aqueous solutions of the synthesized PCL–PEG–PCL (or PEG–PCL–PEG) copolymers can rapidly form the gel in situ after injection under physiological conditions. The hydrogel created shows the ability to control the release of its cargo, made of drugs, proteins and/or gene sequences [52]. As before, the sol–gel behavior can be tuned varying the chemical composition and the polymer concentration and is reversible due to physical crosslinks. Both the diblock and triblock copolymers are instead able to form microspheres or independent NPs with different possible cargos. Thanks to the blocks different properties, all the formulations of this hydrogel had been tested to efficiently encapsulate not only hydrophilic and hydrophobic small-drugs but also macromolecular proteins and genes, releasing them for a sustained period [53]. First studies on this material were done years ago on 5-FU-loaded into PECE triblock copolymer, which shows the same thermosensitive injectable hydrogel properties as PCEC [54]. Interesting properties and therapeutic effects were showed by PEG–PCL diblock copolymer under the shape of nanocarriers for the co-loading of 5-fluorouracil and gene, a DNA sequence (pEGFP), for combination therapy [55]. These systems are polyplexes, constructed by the electrostatic interactions between polymer, drug and DNA. 5-FU among chemotherapeutics is the gold treatment for colorectal cancer as mentioned above; it is an equivalent of uracil in which the hydrogen atom at C5 position is replaced by a fluorine atom, and it can enter the cells thanks to the easy uracil recognition. DNA-loading by electrostatic absorption causes the increase in size and the neutralization of the surface charge, but it maintains stability. PEG chains that are present on the NPs surface are able to avoid the coating of proteins when the nanocarrier is injected into the blood circulation, while the PCL core carries the drug. Alternatively, a nanoparticle for the single encapsulation of doxorubicin (DOX)—made of PCEC triblock copolymer and suitable for intravenous administration—was investigated with acceptable therapeutic results for colon carcinoma [56]. Besides gene combination therapy, many other single and dual drug delivery systems were tested for CRC fighting. An injectable hydrogel constituted by PCEC self-assembled micelles can load the hydrophobic paclitaxel (PTX) and 5-FU at the same time, resulting in a thermosensitive hydrogel able to submit sol–gel transition and to be easily synthesized through a one-step lyophilization method without surfactants [51]. In another work, PCEC hydrogel microspheres were tested to protect camptothecin—another anticancer drug—from hydrolysis and to extend its release time, with the drawback of requesting weekly abdominal injections [57].

More recently, a hybrid solution was presented by Y. Ren et al. [58]. They developed an injectable thermosensitive hydrogel of PCL for the incorporation of oxaliplatin (OXA) and tannic acid (as adjuvant) polymeric nanoparticles. The synthesized system is able to reduce the dose and the cytotoxicity of OXA, thus its side effects on healthy cells for a better standing of the patient. It was prepared through a w/o/w double-emulsion method and is suitable for peritoneal cavity injection at sol state. The presented examples of PEG/PCL materials with their payloads were tested for fighting CRC and its metastases to the liver and to the abdominal cavity (colorectal peritoneal carcinoma) with good results. In particular, the synergistic anticancer effects of gene and 5-FU co-loaded NPs was assessed as the most promising among the innovative approaches of combination therapy developed with this material—followed by the hybrid hydrogel-NPs system—despite the differences in the physicochemical properties of drug and gene. Indeed, the release of DNA was faster than that of 5-FU (the elimination pattern is biphasic), over 80% after 48 and 72 h, respectively, even though the encapsulation efficiency was high enough for both. Probably this is also because DNA is bounded to the nanocarrier outer layer, among PEG chains, while the chemotherapeutic lays inside the particle. This system assures a sustained release that could further ensure better gene transfection after a long time of administration. In vivo test on CRC proved the efficiency and the low toxicity of this therapy and the importance of the synergistic effect of the cargo on the results [55].

Like the materials obtained with PEG/PLA, the PEG/PCL systems allow an efficient encapsulation of drugs with poor water solubility—as PTX, OXA or DOX—avoiding in part their side effects and obtaining releasing periods longer than a week with lower cumulative discharge rate. After NPs, the best hydrogel performance is shown by PCEC instead of its PECE variant; both in situ formed hydrogel scaffolds release their cargo by diffusion and degrade mainly through erosion of the polymeric matrix. Aside from gene-5-FU co-loaded NPs, the other drug delivery systems analyzed allows a quite easy scale-up process, an increasingly sought-after feature in drugs. They have been tested as toxic for CRC cells, but their golden application is for inhibiting its spreading into the abdominal cavity, hindering peritoneal carcinomatosis. After this proof, they can be thought to be also applied for the other metastatic behaviors of our interest in avoiding the damages of the usually implied systemic chemotherapy.

#### 2.4.3. Chitin

Moving to the world of natural polymers, chitin is an amino-polysaccharide of major importance, second to cellulose for quantity produced worldwide. It is a poly(β-(1–4)-N-acetyl-D-glucosamine), namely a modified polysaccharide whose units form covalent β-(1–4)-linkages between N-acetyl-D-glucosamine monomers. Therefore, chitin may be described as cellulose with one hydroxyl group on each monomer replaced with an acetyl amine group. This biopolymer is synthesized by an enormous number of living organisms and occurs in nature as ordered microfibrils forming structural components in the exoskeleton of arthropods or in the cell walls of fungi and yeast. Many other living organisms in the low plant and animal kingdoms produce chitin, which serves functions where reinforcement and strength are required. For industrial processing, chitin is extracted from crustaceans, mainly crab and shrimp shells, but it must be treated and graded in terms of purity and color since residual proteins, pigments and impurities cause a problem for further utilization, especially for biomedical applications. Depending on its source, chitin occurs as two allomorphs: α and β forms, which differ in the packing and polarities of adjacent chains and successive sheets. The former is the one with the most interesting properties, and it could be reproduced (with efforts) in the laboratory [59]. The principal drawback of chitin is its insolubility in water and in common organic solvents, which makes its processing difficult. Hence the N-deacetylated derivative was developed: chitosan. It is straightforward that the higher the degree of deacetylation, the higher its solubility: chitin with 40% deacetylation can be sufficiently dissolved in water, but above 50%, the name is changed in chitosan. The hydrophobicity of chitin arises from its extensive hydrogen bonding between the constituent groups that result in its rigid crystalline structure. The deacetylation also leads to the destruction of the secondary structure of chitin, making it amorphous and useful for some applications [60].

Chitin can easily form fibrils and film structures with optimal strength and low water solubility, making it a good candidate material for human body sutures—already tested—and implants. Moreover, it can be processed into gels, sponges, membrane beads and scaffold forms, producing nanomaterials for tissue engineering, wound healing, drug delivery (for combination therapies of our interest) and cancer diagnosis conjugating semiconductor probes [61]. The efficient example reported in the literature by S. Rejinold et al. [62] sees chitin as a multifunctional spherical nanogel for simultaneous drug delivery and bioimaging; the resulting diameter was less than 100 nm, optimal for cell uptake and proteins encapsulation. The positive attributes of excellent biocompatibility and admirable biodegradability with low toxicity and versatile biological activities have provided ample opportunities for chitin development thought in the sense of drug carrier with slow release potency. Even though chitin and chitosan are very similar for chemical structure and some properties, distinctions in utilization and coupling with drugs are present, thus they are here analyzed as separated materials. Obviously, chitin cannot be used as extracted and treated, but proper shapes must be designed. Owing to the poor solubility due to high crystallinity, chitin hydrogel production is not so straightforward: calcium chloride dihydrate-saturated methanol solvents are needed for initial dissolution, while stirring and addition of large excess of water (or methanol) can bring to the swollen state. The result is a hydrogel with high viscosity, which properties depend both on the degree of N-deacetylation and the molecular weight of chitin, but also on Ca^2+^ ions concentration in the solvent, making it suitable for developing scaffolds, membranes and nanostructures [63,64].

**Figure 4:**
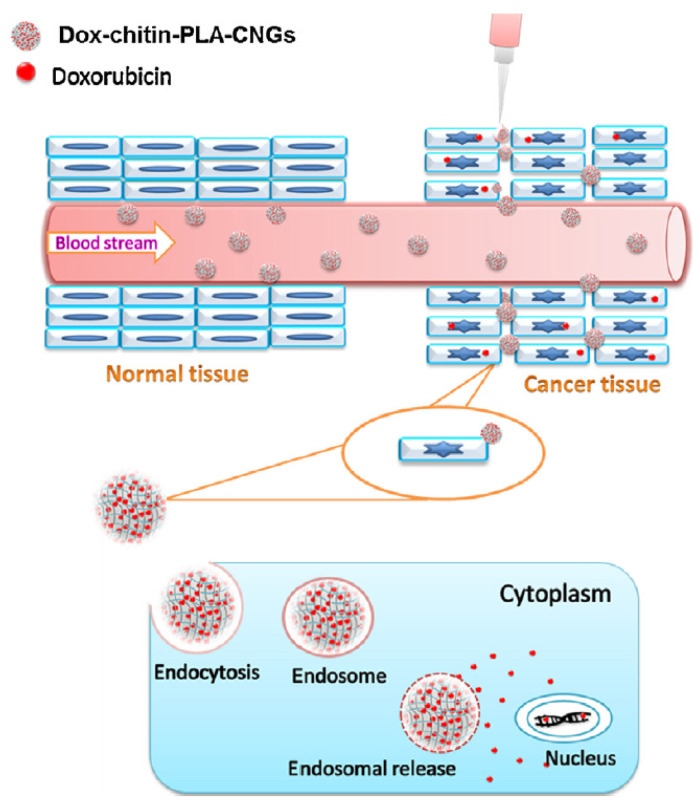
Schematic hypothetical mechanism of doxorubicin (DOX) delivery from chitin nanogels within the cell. Reprinted with permission from Elsevier [69].

The most used shape in the biomedical field is that of biodegradable chitin nanogels, synthesized through a controlled regeneration method. Nanogels are hydrogels confined to nanoscopic dimensions having many attractive properties like size tunability, large surface area, permeability, excellent drug-loading capacity, controlled release and responsiveness to environmental stimuli. The synthesis plans to treat chitin solution with methanol until gel regeneration, then wash several times with water, centrifuge and sonicate for complete removal of methanol and Ca from the nanogel network-like structure—even though the residual CaCl_2_ entrapped is believed to help in crosslinking the polymeric chains of chitin [65]. The main properties of chitin nanogels are water retention, resembling the three-dimensional structure of the native ECM and swelling the most at acidic pH, improving biodegradability thanks to the lysozyme attack, which can specifically cleave the glycosidic linkages. Nanogels can be further modified for targeted delivery by chemically conjugating active moieties for ligand–receptor interactions; however, passive targeting exploiting the EPR effect could be easily achieved thanks to the poor recognition by MPS due to the final hydrophilicity of the gel [66]. Applying this technique to the treatment of colorectal cancer, the principal cargo found in literature is the hydrophilic doxorubicin. Chitin can be coupled with hyaluronic acid (HA) and encapsulate cystamine and DOX to obtain a synergistic effect, safe for intravenous administration: chitin could make HA slowly degrade, and HA is a ligand for the CD44 receptor that is overexpressed in CRC cells. The cross-linked nanogel system is constituted by an amide bond between the HA carboxyl group and the amine one placed at one end of cystamine, while the unreacted amine group of cystamine undergoes ionic interaction with the hydroxyl group of chitin; DOX is physically adsorbed onto the structure [67]. A possible alternative was to encapsulate DOX in chitin only, leaving the mixture stirred for the proper incubation time; the nanogels obtained had a spherical morphology and showed significant toxicity and cancer cell uptake [68]. Or again, a solution could be to regenerate chitin with PLA (PLLA specifically) and load DOX as before: the nanogel obtained will be thermally stable for blood administration and will achieve passive targeting. Moreover, the drug could be released in endosomes or lysosomes by pH-controlled hydrolysis (Figure 4) [69]. Besides nanogels, for the treatment of CRC, chitin can be found in literature as nanoparticles constituted by its amorphous form. Thus, complexation with TPP is needed for creating crosslinks, like for the weaker structure of chitosan. This kind of formulation was tested to load paclitaxel (PTX)—that is hydrophobic—with a quite good encapsulation efficiency for causing cancer cell death through apoptosis [70]. The presented solution is suitable for passive targeting of CRC through EPR effect, while the functionalization with epigallocatechin-3-gallate (EGCG, bioactive polyphenol found in green tea) has brought to liver active targeting.

This system was able to carry honokiol, another hydrophobic chemotherapeutic, by ionic interactions maintaining the spherical morphology. EGCG was able not only to provide active targeting but also to have a synergistic role together with honokiol for contrasting different stages of cancer [69].

Analyzing neatly the structures presented, the principal shape is that of injectable nanogel or nanoparticles, more physically stable in physiological fluids with respect to liposomes and micelles. Chitin-HA nanogels may undergo some clearance due to protein surface binding, but the nanosized formulation is able to enhance the availability of the chemotherapeutic agent at the tumor site, obtaining a 2-day long sustained release of DOX, also thanking the active targeting provided by HA [67]. DOX in the cancer cell is thought to interact with DNA by intercalation and inhibition of macromolecular biosynthesis, hindering its replication [68]. Among DOX-loaded nanogels, chitin-HA ones revealed higher suitability for treating primary CRC, while chitin alone and chitin-PLLA formulations have shown toxicity predominantly against metastatic liver spots of I and II stage. Chitin degradation in the colon is caused by anaerobic microbes that have the ability to break the glycosidic linkages of the polymer leading to chain destruction into monomers. This polymer has the advantage of not be digested neither in the stomach nor in the small intestine, leading to maximum drug release in the colon whenever the administration would be oral. Whereas drug release for intravenous administration—like the ones aforementioned—happens after cell endocytic pathway uptake usually by swelling and diffusion triggered by acidic pH present in lysosomes: ionization of pendant groups causes electrostatic repulsion in the nanogel network, thereby resulting in the enlargement of the meshes and allowing the excess solvent influx. The main drawback seen in using chitin is the implication of toxic solvents for its transformation in hydrogel and nanoparticles, principally for the latter. However, no side effect on the major organs was observed during the testing of the mentioned formulations. In the end, studies to transform chitin into a stable implantable scaffold are currently taking place: chitin has strong mechanical properties that can make it suitable for this task. However, users and researchers must keep in mind that chitin and chitosan derive from fungi, but also from animals, mainly from shellfish: their usage implies breeding and sacrifices, thus not so sustainable and renewable. The alternative—valid only for α-chitin—is in vitro production through biosynthesis.

#### 2.4.4. Chitosan

Chitosan (CS) is a semicrystalline amino-polysaccharide prepared by the aforementioned chitin deacetylation. Hence it is a natural polymer, but not cellulose-like, considering the presence of four elements in its formula, its cationicity, its film-forming ability and polyelectrolytes complexes-forming capacity. Chitosan units are primary aliphatic amines that can be protonated by selected acids; the resulting salts are water-soluble [71]. CS has been widely utilized in the biomedical field—as hereon presented—thanks to its nontoxicity, good biocompatibility, biodegradability via enzymatic depolymerization and permeation enhancing properties. The CS hydrogel formulation can be an efficient drug delivery system for substituting intraperitoneal, oral or blood chemotherapy. The CS aqueous solution does not possess a thermosensitive feature, but the addition of β-glycerophosphate (β-GP) solution directly modulates the hydrogen bonding and electrostatic interactions between polysaccharide chains to form a gel, whose properties can be tuned with CS degree of deacetylation [72]. Hydrogels obtained by CS and β-GP are injectable solutions that turn into a non-flowing gel around body temperature because of three types of interactions: electrostatic between the ammonium group of CS and the phosphate one of β-GP, H-bonding and hydrophobic interactions between chitosan chains owed to the lower repulsion in the presence of β-GP [73]. The time of gelation is higher than that of hydrogels based on synthetic polymers, and the process is triggered by temperature and not by pH. Moreover, β-GP is essential for the sol–gel transition to happen. This slow evolution allows a longer shelf life of the sol state at low temperatures, showing no changes in viscosity for three months. There are many studies in the literature involving chitosan hydrogels as drug delivery systems. The ones applied to our goal imply thermosensitive CS hydrogel that load 5-FU, alone or together with cisplatin. The loading of 5-FU only brings to a formulation that gels reversibly in 8 min under physiological conditions, making it suitable for intraperitoneal or intratumoral injection [74]. Dual drug delivery systems are more recent; in this case, the co-loading of 5-FU and cisplatin was analyzed: it can efficiently inhibit colorectal cancer growth and metastases. This formulation guarantees a slow and sustained drug release and an increased drug uptake [72].

The hydrogel is not the only shape that chitosan can assume; it could also be used for constituting nanoparticles or for coating them to enhance biocompatibility. Since chitosan–chitosan interactions are too weak to form crosslinks, as reported, the formation of chitosan NPs (nanogels) is unthinkable. For this reason, the only way to obtain spherical complexes is to ionically crosslink chitosan to tripolyphosphate (TPP) and obtain polymeric NPs useful for drug-carrying. The main advantage lays in the facile crossing of biological barriers and good targeting thanks to chitosan biocompatibility, even when the NPs are injected into the blood circulation. CS/TPP NPs show a labile behavior and fast disintegration as soon as they experiment with the body environmental conditions and biological fluids incorporation, but when they are loaded with macromolecules or drugs, the interactions between them and the gel network can effectively make particles much more stable [75]. The master technique involving chitosan for reducing the number and the volume of CRC liver metastasis foci sees the natural polymer complexed with TPP and interleukin-12 (IL-12) in the shape of nanoparticles [76]. IL-12 is a multifunctional cytokine that enhances helper T cells differentiation and proliferation of natural killer cells, but its intravenous administration has proven to be excessively toxic; it seemed thus useful to exploit the spontaneous complexation with chitosan. The ionic interaction between CS and TPP reduces the binding capacity of opsonins, escaping the MPS clearance: this system was produced to be injected into the blood circulation. Liver passive targeting of these NPs is dependent on the diameter (100–200 nm maximum) and physicochemical properties; the NPs target liver Kupffer cells and release the entrapped drug thanks probably to the low pH of tumor sites.

An interesting variation to achieve a direct active targeting of the dendritic cells which reside within the tumor was to functionalize chitosan with mannose, producing mannosylated chitosan injectable NPs prepared to induce mannose receptor-mediated endocytosis [77]. Moreover, other possible cargos could be IFN-γ, coupled with IL-12, or directly doxorubicin and a gene, like a siRNA sequence [78]. Once again, a cytokine cargo for immunotherapy can be seen as successful prevention of cancer hepatic spreading.

The last use of chitosan, concerning colorectal cancer metastasis fight, sees it as a *coating* for NPs to better minimize opsonization and facilitate passive targeting, protecting the chemotherapeutic cargo (usually 5-FU or paclitaxel). Two examples are those reported by K. M. Kamel et al. [79] for coating solid lipid nanocarriers based on a core of cinnamon/oregano derivatives and by J. Kanwar et al. [80] as the first coating of ceramic nanoparticles; in this case, chitosan could be covered again by alginate, and the system resulted suitable for oral administration. The release model of the CS/β-GP hydrogel cargo is thought to occur mostly by diffusion but could be accelerated by the weight loss of the gels due to polymer degradation. Macromolecules discharge can be sustained over a period of several hours to a few days, while incorporation of nanoparticles is suggested for achieving a continuous release over more than one week. The hydrogel single encapsulation of the backbone chemotherapeutic treatment 5-FU was developed principally to fight intraperitoneal CRC metastases, while the dual drug system—5-FU combined with cisplatin—can efficiently inhibit CRC angiogenesis and metastasis behavior to the liver and lungs. Tumor volume measurements showed comparable efficacy of 5-FU administered alone in gel or through commercial blood or intraperitoneal injection, with a greatly improved safety profile of the former [74]. The formulation with both 5-FU and cisplatin instead is efficient in hindering the CRC diffusion through the portal vein to the liver, as demonstrated by Q. Yun et al. with studies in vivo [72]. Furthermore, qualitative and efficacy studies were done to compare chitosan with other hydrogels formulations—as PCL–PEG–PCL—the results are interesting: chitosan is a natural multifunctional polymer that can achieve large-scale production and is more effective against metastases. Instead, when dealing with CS/TPP NPs, pH can control the swelling of the particles, triggering the cargo release into the most acidic regions, which are endosomes or lysosomes when the administration is intravenous; on the contrary, salts, meeting provokes the disintegration of the carrier. The most promising technique involving chitosan for fighting liver CRC metastases is constituted by CS-TPP/IL-12 nanoparticles: the studies carried out firmly have demonstrated good apoptosis of the cancer cells and an anti-angiogenesis effect. It must be underlined that often surgery paradoxically enhances metastasis development inducing immune deregulation, meaning that a combined immune and chemotherapeutic intervention is needed: CS-TPP/IL-12 NPs can be a suitable technique.

#### 2.4.5. Alginate

Polysaccharides that are precisely activated by the physiological environment of the colon hold great promise as they provide improved site-specificity and meet the desired therapeutic needs. Alginate can be considered one of these. Alginate is a linear and anionic polysaccharide derived from brown seaweed, it is composed of alternating blocks of α-1,4-L-guluronic acid and β-1,4-D-manurunic acid units, and it is available in various grades. Although it can be produced by bacterial sources, it is commercially available from algae in the form of salt, i.e., sodium or calcium alginate [81]. Among all the properties, alginate is useful for colon targeting for its remarkable crosslinking capability, pH-sensitivity and mucoadhesiveness. The biodegradability, low toxicity, and chemical versatility of alginate are well known, but its unique property to form stable gels in aqueous media and mild condition by addition of multivalent cations makes this biopolymer very useful for drug delivery and cell immobilization. In particular, it can be exploited for microencapsulation and coating techniques together with other polymers, but also as hydrogel microparticle or scaffold owing to the sustained release characteristic suitable for colon-targeted delivery. Alginate matrix can be prepared, after purification, to reach biocompatibility through physical and/or chemical crosslinking of the polymer chains, with cations coupling or through hydrogen/covalent bonds, respectively. The presence of a counter-ion—like calcium—is necessary since alginates do not gel alone because of the rigid poly(L-guluronic acids). Alginate gelation takes place when divalent cations (usually Ca^2+^, even though it does not show the highest interaction strength) interact ionically with blocks of guluronic acid residues, resulting in the formation of a 3D network that is usually described by the “egg-box” model [82].

pH-sensitive hydrogels are thus formed in mild conditions, without requiring heating and cooling cycles thanks to the solubility of alginate in water. The tendency in the biomedical application is that of exploiting ionic crosslinking since it offers adequate methods for the entrapment of substances, preserving biological activity. Sometimes both types of crosslinking are used for enhancing mechanical properties when dealing with injectable hydrogels or scaffolds for cell encapsulation [81]. Owing to the presented properties, alginate is an attractive material for *oral* colon-targeted drug delivery systems. The colonic region is the least hostile environment of the GI tract, and oral administration is often preferred by patients because of its non-invasive nature: alginate microparticles are able to protect the cargo to the colon thanks to pH-sensitiveness. The presence of carboxylic groups confers to the polymer the sensitivity to external pH stimuli: at low pH, typical of the stomach, alginate forms an insoluble structure, while at pH near the colonic one (pH = 6.8–7.4), the expansion and swelling of the hydrophilic matrix are maximum due to an increase in repulsion forces, triggering cargo release. Moreover, alginate is classified as a good mucoadhesive agent, which might help in prolonged adhesion of the drug in the intestinal mucosa as a result of its residence time in the colon [81]. Indeed, the most spread application of alginate—as reported in the literature—consists of microparticles, namely polymeric beads, microcapsules, microspheres of 1–1000 μm enclosing bioactive substances, for oral administration of colon therapies. Plenty of studies are interested in testing this ability and bringing the product to the market, experimenting with many different alginate-based structures. The one that deserves more attention for our purpose is presented by B. Zhang et al. [83] regarding the hindrance of colon cancer liver metastasis after CRC resection. The system is constituted by graphene oxide-based sodium alginate functionalized microparticles (2 μm particle size) loaded with 5-FU as an anticancer drug; it is thus able to effectively deliver the drug to the colon and maintain a sustained release of the chemotherapeutic agent. Alternatives are represented by alginate *beads*, like the Ca-alginate formulation reported by A. Sookkasem et al. [84] loaded with curcumin and coated by Eudragit^®^ (copolymers derivatives of acrylic and methacrylic acid esters with pH-dependent solubility), or the one of F. Hsu et al. [85] regarding calcium pectinate-alginate microspheres coated with Eudragit and carrying cisplatin. Again, the system presented by T. Agarwal et al. [86] made of calcium alginate-carboxymethyl cellulose can bring the usual 5-FU cargo. In the last proposal, the gelation occurs through the ionic method: anionic carboxyl groups present in alginate and carboxymethyl cellulose interacted with bivalent calcium ions to form the gel. The result has combined the approach of pH-dependent and microbially triggered drug release. Also, in this case, active targeting can be boosted by binding folic acid for the exploitation of folate receptor recognition of CRC cells; a valuable formulation is that of D. Bansal et al. [87]: folic acid conjugated liposomes encapsulating oxaliplatin entrapped in alginate beads and coated furtherly with Eudragit^®^. The other typical shape to imply alginate for oral administered colon-targeted devices is that of microcapsules. Eudragit^®^ coated, and indomethacin loaded nanoparticles can be encapsulated into alginate for assuring colon-specific delivery, as reported by Y. Ma et al. [88] with a resulting 2 mm alginate pellets to swallow. A. Abbaszad Rafi et al. [89] instead followed a completely green and environmentally friendly route to prepare pH-sensitive drug carriers for colon-specific delivery; in particular, they have synthesized alginate microcapsules with two layers of coating—made of chitosan and carboxymethyl cellulose, oppositely charged biopolymers—to incorporate naproxen and magnetic NPs. The resulted particles overcame the drawbacks of alginate like porosity and burst drug release. Moreover, they could be driven by means of an external magnet to the target site. Like in previous techniques, further studies are required to characterize the releasing method in vivo. Alginate can be applicable also as a suitable in vitro 3D tumor model to study different aspects of cancer cell behavior. Hydrogels are indeed important class of biomaterials as they could mimic the ECM with its structural architecture, composition and biological functions; this aspect, beside tissue engineering, can be useful for cancer research approaches, limiting animal-based experiments. Alginate 3D scaffold was investigated for mimicking the tumor microenvironment and screening the effects of chemotherapeutics like 5-FU or curcumin on colorectal cancer cells [90].

**Figure 5:**
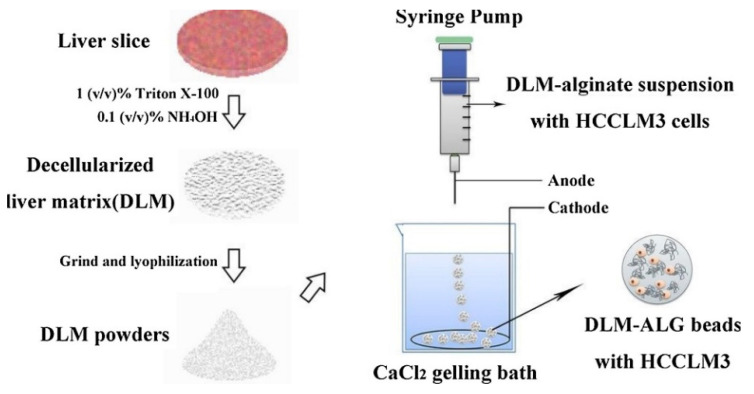
Schematic illustration of the preparation of decellularized liver matrix (DLM) and decellularized liver matrix–alginate hybrid gel beads (DLM–ALG beads). Reprinted with permission from Elsevier [92].

The efficacy of alginate blends—with gelatine, for instance—was tested too for enhancing crosslinking properties [91]. In order to study the CRC metastases, liver matrix–alginate hybrid gel beads were constructed: it efficiently mimicked liver environment and HCC metastases to screen therapeutic drugs more easily (Figure 5) [92,93]. After being cultured in liver matrix–alginate beads, HCCLM3 cells showed higher cellular viability and metastatic potential with respect to those cultured in conventional alginate beads. Thus, liver matrix–alginate beads have high potential as a novel 3D culture system for exploring the mechanism of tumor metastasis and screening antimetastatic drugs.

The future alginate applications forecast biodegradable hydrogel scaffolds implantation in the tumor resection site for the extended-release of perioperative immunotherapy to overcome the transient immunosuppressive state associated with wound healing after surgery, a situation that promotes tumor recurrence and distal metastases. It is not science fiction, but solid bases of current studies confirm good promising results that can overcome the issues related to systemic or local immunotherapy. The cargo of the mentioned scaffold comprehends immunostimulatory compounds—such as antibodies, cytokines, interferons and agonist of innate immunity (R848 or STING-RR)—aimed to activate both the innate and adaptive arms of the immune system, turning the post-resection microenvironment into immunostimulatory. This technique was first applied to breast cancer, but it is also promising for those cancers with high mortality and probability of distal metastases as CRC. The correct mechanical properties of alginate hydrogel are, however, still under study. Thanks to the optimal results of tests presented in the literature, pH-responsive alginate microparticles lead to the opportunity for designing targeted oral colon delivery systems with a biomimetic approach of variation of pH value through the GI tract in the human body. It is the ion exchange process between sodium and calcium ions that are supposed to be responsible for the swelling and subsequent degradation of alginate in the colon. Despite their poor encapsulation efficiency, the most promising results were obtained with graphene oxide-alginate-5-FU microparticles, which significantly inhibited tumor growth and liver metastasis with prolongation of survival time thanks to the correct targeting and good biocompatibility: the drug is released outside the cells and enters by diffusion, no need to design microparticle cell uptake [83]. All the other formulations of beads and microcapsules are equally capable of preventing drug release in the upper GIT and discharging it upon the arrival in the colon thanks to pH sensitivity and microflora degradation, triggering tumor cells apoptosis, hence targeting the primary colorectal carcinoma. Alginate can often be combined with other materials (with the abovementioned chitosan, for instance) for enhancing properties in specific applications: the combination forms of polysaccharides or the chemically modified ones eliminated the drawbacks associated with the use of a single polysaccharide. In conclusion, alginate can be used effectively for constituting beads, scaffolds and 3D matrices that resemble the TME.

The main administration under the current experimental test is the oral one, but implantable, biodegradable hydrogel scaffolds for repairing immune deficiency caused either by the tumor itself or by surgery with an immunotherapeutic cargo are very promising but still in a preclinical trial.

#### 2.4.6. Hyaluronic Acid

Hyaluronic acid (HA) is another natural polymer widely used in the biomedical field. It is an anionic linear biopolymer composed of alternating disaccharide units of D-glucuronic acid and N-acetyl-D-glucosamine with β(1–4) interglycosidic linkages [94]. More than 50% of HA in the human body is present in the skin, lung and intestine, while minor quantities appear in the umbilical cord and blood. Currently, HA is commercially produced from animal tissues such as cockscomb and from microbial fermentation, therefore—like chitin and chitosan—it is not so renewable, but this explains its biocompatibility. HA functions are associated with the regulation of cell behaviors since a strong correlation between its presence, and cell migration and proliferation has been demonstrated: HA plays pivotal roles in wound healing, cell motility, angiogenesis and ECM construction. The tasks of this important polymer depend on its molecular weight: high MW HA maintains cell integrity and water content in ECM, while degraded fragments are known to induce receptor-mediated intracellular signaling [95]. HA follows the usual route of degradation and excretion: it is taken up in tissues and degraded by lymphatic systems, then the degraded HA enters the blood and is transported to the liver where it is catabolized.

**Figure 6:**
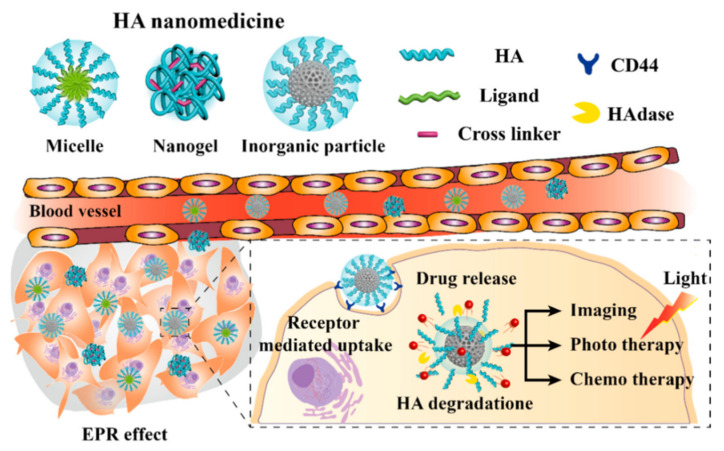
Schematic representation of the advantages and applications of hyaluronic acid (HA). EPR: enhanced permeability and retention; CD44: cluster determinant 44. Reprinted with permission from MDPI [98].

Intermediate degradation products are guluronic acid and N-acetylglucosamin, which are further decomposed to CO_2_, H_2_O and urea. The physiological turnover of HA is remarkably rapid: the half-life of injected HA in plasma is around 4 min, but longer circulation time can be achieved through modifications such as chemical attachments of pendant groups or crosslinking for the preparation of hydrogels [96]. Recent studies have assessed high HA levels as an index of sinusoidal obstruction syndrome—present in > 50% of the patients—due to oxaliplatin-based chemotherapy for patients with CRC liver metastases that have submitted one or more hepatectomies [97]. Inspiring by this natural feature and thanks to its biocompatibility, biodegradability, non-immunogenicity, non-inflammatory and nontoxicity properties, HA is a suitable candidate for our purposes. The principal shape of HA as a drug carrier is that of the injectable hydrogel, either thermosensitive or for intravenous administration. The protocols to prepare HA hydrogels can be classified into three types: direct crosslinking of HA, crosslinking of HA derivatives or crosslinking between two different kinds of HA derivatives. The first and the second possibilities imply the usage of an external crosslinker, while the third method exploits Michael-type addition or click chemistry and is suitable for forming hydrogel systems in situ [96]. Injectable hyaluronic acid hydrogels can be thermosensitive if the administration is local (intraperitoneal or intratumoral) and a formation of a drug depot after a sol–gel transition is required, while the shape of nanogels or little particles made of crosslinked polymers are preferred for intravenous administration. In order to obtain gelation, another polymer of opposite affinity must be present for the generation of micelles, which can self-assemble, whereas in the second case, a crosslinker is needed to form the nanogel bead. Here the worlds of hydrogels and NPs are coupled together, as already seen for other materials. Intravenous administration involves nanometer-sized HA nanomedicines, which can selectively deliver drugs or other molecules into tumor sites through EPR effect, but HA can also interact with overexpressed receptors in liver and colon cancer cells such as cluster determinant 44 (CD44) or receptor for HA-mediated motility, exploiting active targeting for being internalized (Figure 6). Moreover, HA can be degraded inside the cell by a family of enzymes called hyaluronidase to release directly drug or molecules; this kind of degradation can be exploited also with thermosensitive hydrogels, releasing the cargo outside the cell. Enhanced antitumor efficacy can be achieved via stimulated-drug release, such as redox reactions or exposure to an acidic pH [98].

Nanoparticles for intravenous administration can be applied in primis to early colorectal tumor detection, for example, conjugating PEG-HA copolymers to an imaging dye as Cy-5. Owing to HA active targeting, NPs accumulate in the cancer site and are detected through a non-invasive near-infrared fluorescence tool, enhancing diagnostics of small-sized colon tumors as well as liver metastases. The same formulation was used to attach irinotecan to the hydrophobic core of the particle for targeted chemotherapy [16]. In addition to diagnostics, there are many HA-based techniques for drug delivery aiming to improve efficacy, safety and cytotoxicity. The one reported by You-Sin Jian et al. [99] studied a HA carrier loaded with nimesulide, a powerful cytotoxic drug that can be released intracellularly through CD44-mediated endocytosis. The systems showed in vivo selective accumulation in the colorectal tumor area, triggered by CD44 overexpression. Another administration route that involves nanoparticles (nanogels, beads) is the oral one, similarly to alginate. With this purpose, HA-chitosan coupled nanoparticles were synthesized with an oxaliplatin cargo and a Eudragit^®^ coating: the system is resistant to the upper GI tract fluids, while swells due to the lower pH in the colon releasing the majority of the drug encapsulated [100]. In the mentioned application, chitosan had to be complexed with TPP to retain a shape, while oxaliplatin was loaded into CS/TPP NPs, and these were encapsulated into HA beads, coated in turn with Eudragit^®^. The system demonstrated positive colorectal tumor targeting. Often local administration is convenient, hence in recent years, injectable thermosensitive HA hydrogels are spreading their implication as efficient carriers for CRC combination therapies. The most important progress made in this field concerns immunotherapy: HA was conjugated with tyramine and loaded with IFN-α2a, producing an injectable gelling hydrogel able to inhibit the proliferation of liver cancer cells, inducing apoptosis and hindering angiogenesis [101].

Other systems involving HA injectable thermosensitive hydrogel were tested for chemotherapy and hindering intraperitoneal adhesions caused by CRC resection during surgery. The formulation proposed by J. Luo et al. [102] concerns combined chemotherapy: 5-FU, cisplatin and paclitaxel incorporated into PCEC microspheres were integrated into a hyaluronic acid hydrogel. The direct injection into the abdominal cavity allowed to administer the drugs intraperitoneally, shielding local intestinal infiltration of the tumor cells and thus preventing colorectal peritoneal carcinomatosis and liver/lung metastases. 5-FU and cisplatin were released first due to HA hydrogel absorption, followed by a slower release of paclitaxel because of PCEC microsphere degradation. Instead, C. Chen et al. [103] proposed a composed hydrogel for the prevention of peritoneal adhesions in the abdomen, a consequence of tumor resection. The system was synthesized with the union of HA, chitosan and poly(N-isopropylacrylamide): sol at room temperature; it exhibits sol–gel transition around body temperature.

Combination of adhesion prevention and chemotherapy was finally proposed by J. E. Lee et al. [104], conjugating HA with carboxymethyl cellulose, loading oxaliplatin and tuning the microstructure, the rheology and the degradation behavior, offering a novel formulation for a double purpose.

The last quite spread use of HA implies tissue engineering, only cited here since not central for liver CRC metastases treatment. In this field, HA has great potential due to its unique biological features—mainly swelling response, long-term stability and enzymatic degradation—and it is often accompanied by alginate to obtain biocompatible and biodegradable hydrogel matrices with tailored properties, suitable for cellular ingrowth. Controllable biodegradability can facilitate angiogenesis, osteointegration and cell phenotype preservation [105]. M. M. Perez-Madrigal et al. [106] recently reported an injectable click-hydrogel, formed in situ following the fast and simple thiol-yne click chemistry, suitable as a 3D scaffold to support and promote soft tissue regeneration, like cartilage. The mechanical strength is supported by HA, while alginate gives flexibility due to physical crosslinks with Ca^2+^ improving viscoelastic properties. Gelation occurs in 5 min. Nanoparticles and nanogels for intravenous administration allowed early detection and targeted therapy: HA hydrogels can improve current techniques that have a high miss rate of colon cancer leading to metastases. In this form, hyaluronic acid wanted to resemble alginate for oral administration, the concentration at the site of action and the exposure time was high and the formulation allowed to enhance antitumor efficacy, but the mainstream for this kind of administration remains alginate. In the beginning, HA was put aside owing to its high clearance rate from the body that should imply frequent infusions, resembling those of classic chemotherapeutics. After the discovery of HA gelling properties, the shape of hydrogel began to be studied as active agent depots and protection, directly injectable in situ. The tests regarding HA for fighting intraperitoneal post-surgical adhesions and for treating CRC through intraperitoneal/intratumoral chemotherapy are many: it has become promising as a drug carrier with slow-release potency under the shapes of injectable thermosensitive gel or 3D crosslinked hydrogel scaffold. The advantages of these technologies are the enhancement of local concentration and retention prolongation of the effective drug concentration at the site of action, leading to a decrease in dose frequency. Tissue engineering has inspired 3D implantable HA scaffolds for drug delivery directly in the site of tumor resection, with the possibility to locally deliver immune and chemotherapies, similarly to what previously shown with alginate biodegradable hydrogel scaffolds: to this end, HA implants due to their unique degradation kinetics may achieve similar immunostimulatory results obtained with alginate [107]. The results obtained with different models of spontaneous metastasis showed that the prolonged release of agonists of innate immunity from HA-based hydrogels allows obtaining better results with respect to the ones obtained with their systemic administration. The hydrogel can guarantee proper degradation rate and release of the agonist in vitro and in vivo.

## 3. Challenges and New Perspectives

Beyond the advantages discussed, some challenges must be solved in order to make feasible the translation to the clinic, and subsequent commercialization of the devices described. First of all, deeper investigations on possible toxic effects should be done to improve their biocompatibility. Because of this, many preclinical studies are needed, investigating the immune system interactions and unanticipated toxicities. Second, their target activity is a pivotal point, and improving the specificity of the functional formulation is essential. Then, the preservation of the pharmacological activity when binding with the target should be maintained. In this framework, nanodrug structure design and fabrication protocol are essential, considering that many biological mechanisms related to materials’ effects on the human body are still largely unknown, and because of this, clinical efficacy studies are required. Beyond pharmacological activity and studies on possible toxic products derived from NPs, key importance should also be given to technical issues and, in particular, the manufacturing method, which up to now represents one of the main challenges in device translation to the clinic. Indeed, scale-up from a few grams produced in the laboratory to several kilos on an industrial setup is required. Therefore, reproducible, easily scalable processes following the good manufacturing practice (GMP) principles are important prerequisites. The facility of scale-up production and the control over critical design features are also extremely important for a quick translation to clinics.

## 4. Conclusions

Liver metastases are one of the major concerns following primary colorectal cancer and its resection. In addition to a brief overview of classic treatments, liver- and colon-targeted delivery systems developed in the last decade have been presented divided for material implied. Many different materials can be combined with one another, as mentioned, to achieve better performances. Both synthetic and natural polymers have been developed, together with diverse bio-adhesive colon-targeted drug delivery systems that use polysaccharides as drug carriers, as shown above.

Among the cited materials, alginate and hyaluronic acid are the most hopeful candidates under preclinical testing. The shapes, materials and degradation kinetics are under extensive investigation in order to achieve optimal results for each specific application by the use of temporal and anatomical controlled delivery systems based on hydrogels and nanoparticles.

While preformed hydrogel scaffolds are often discouraged by the need for invasive surgery requested for the implantation, for patients undergoing surgical resection of colorectal cancers may represent a unique treatment opportunity window to further control local cancer recurrence and metastatic spreading to other distant organs such as the liver. Due to this, the majority of the proposed engineered materials are shaped as injectable hydrogels, nanogels or nanoparticles, which forecast other routes of administration—like intravenous, intraperitoneal, abdominal and seldom oral (Table 1). In the future, we envision a particular interest in implantable hydrogel scaffolds as immunotherapy carriers and adjuvants, but also as part of complex combination therapies that may promote strong antitumoral effects.

## Figures and Tables

**Table 1:** Different delivery systems, divided for material implied, aimed to hinder hepatocellular carcinoma and colorectal cancer metastatic behavior. Here examples of possible cargos are reported, together with some remarks for every type of biomaterial, showing some pros and cons.

Material	Formulation	Cargo	Remarks	Ref.
*PEG/PLA*	Injectable thermosensitive hydrogel made of micelles	Cytokines and Toll-like receptor agonists	Hydrogel undergoes sol–gel transition at physiological conditions entrapping the immune and chemotherapeutic cargo. Minimal cytotoxicity and hemolysis. Cargo release through swelling diffusion in 7 days and hydrogel disappearance in 10 weeks. Empty hydrogel prevents intraperitoneal post-surgery adhesion, and the vaccine-loaded one improves HCC survival by 20%.	[42,43,44,45,46,47]
	Nanovesicles	Doxorubicin HMME Curcumin	Injected in blood vessels, they can reach the target thanks to the EPR effect or active targeting with Fol. Satisfactory liver CRC metastases targeting hydrophilic and light-sensible hydrophobic chemotherapies.	
*PEG/PCL*	Nanoparticles	5-fluorouracil and DNA	PEG–PCL polyplexes constructed by electrostatic interactions, injectable in blood vessels. Combined and effective gene and chemotherapy, with synergistic effects against CRC. Optimal efficiency encapsulation, 80% drug release in 72 h and poor MPS recognition thanks to PEGylation.	[48,49,50,51,52,53,54,55,56,57,58]
		Doxorubicin	NPs injectable in blood vessels, good results against CRC thanks to passive targeting and DOX slow release.	
	PCEC hydrogel	5-fluorouracil and paclitaxel	Injectable in situ, sol–gel transition occurs at body temperature. Applied mainly for inhibiting CRC spreading into the abdominal cavity, hindering peritoneal carcinomatosis (CRPC).	
	PCEC microspheres	Camptothecin	Hydrogel layer to protect the cargo from hydrolysis. Weekly abdominal injections are needed. Main application: counteracting CRPC.	
	PCL injectable thermosensitive hydrogel + NPs	Oxaliplatin and tannic acid	Hybrid solution: PCL scaffold with NPs encapsulated. The system undergoes sol–gel transition at physiological conditions, shielding the toxic effects of its cargo. Assessed for CRPC therapy, but under study for liver CRC metastases.	
*CHITIN*	Nanogels	Doxorubicin	Chitin can be coupled with hyaluronic acid or PLA to obtain injectable nanogels for intravenous therapy. Technique useful for both CRC and HCC foci: the drug is released in endosomes or lysosomes by pH-controlled hydrolysis.	[59,60,61,62,63,64,65,66,67,68,69,70]
	Nanoparticles	Paclitaxel Honokiol	Injectable systems whose integrity is provided by complexation with TPP. Passive targeting can be substituted by the active one binding EGCG to chitin. PTX-loaded NPs are more active against CRC, while honokiol ones are effective against HCC.	
*CHITOSAN*	CS/β-GP injectable thermosensitive hydrogel	5-fluorouracil 5-fluorouracil and cisplatin	Reversible hydrogel suitable for intraperitoneal, abdominal or intratumoral injection that shields the toxicity of the cargo. The combined therapy is very efficient in hindering CRC metastatic spreading to the liver.	[71,72,73,74,75,76,77,78,79,80]
	CS/TPP nanoparticles	Interleukin-12	Immunotherapy is injected into blood vessels. NPs reach the hepatic tumor site through passive targeting, and the release of the cytokine is triggered by pH. It is the most effective treatment for liver CRC metastases among the chitosan alternatives.	
*ALGINATE*	Microparticles	5-fluorouracil	Suitable for oral administration and colon targeted delivery, thanks to pH-sensitivity. Graphene oxide-based sodium alginate functionalized microparticles for the specific treatment of liver CRC metastasis.	[81,82,83,84,85,86,87,88,89,90,91,92,93]
	Beads or microcapsules	Curcumin Cisplatin 5-fluorouracil Oxaliplatin Indomethacin Naproxen	Suitable for oral administration and colon targeted delivery, thanks to pH-sensitivity. The main counter-cation used is Ca^2+^, alginate can be complexed with carboxymethyl cellulose, pectinate or chitosan; the system can be coated with Eudragit^®^, encapsulate liposomes or magnetic NPs for external control of the position. Active targeting of CRC cells is achievable by conjugating folic acid.	
	3D hydrogel matrices	Colon or liver cancer cells	Alginate 3D scaffolds can efficiently mimic the TME and allow the possibility to screen the effects of new chemotherapies, limiting animal-based experiments.	
	Biodegradable hydrogel implant	Immunostimulatory compounds	Alginate scaffold-loading antibodies, cytokines, interferons or immune system cells can be placed into CRC resection site during surgery to prevent cancer recurrence and distal metastases, to the liver, for instance.	
*HYALURONIC ACID*	Nanogels and nanoparticles	Irinotecan Nimesulide	Nanogels are suitable for intravenous administration. HA can be used alone or paired with PEG, conjugating dyes an early primary CRC and metastases diagnosis can be achieved thanks to the CD44 active targeting promoted by HA specificity.	[16,94,95,96,97,98,99,100,101,102,103,104,105,106,107]
		Oxaliplatin	HA-CS/TPP beads coated with Eudragit, suitable for oral administration and colon targeting.	
	Injectable thermosensitive hydrogels	IFN-α2a 5-fluorouracil, cisplatin, paclitaxel Oxaliplatin	Administration in situ thanks to the gelling property of the material. One week to one-month degradation can be achieved by tuning rheologic properties. In addition to chemotherapy and peritoneal adhesions prevention, immunotherapy is the future application of HA hydrogels for CRC treatment and metastases hindrance.	
